# Modified coconut shell biochars (MCSBCs): Fabrication and their adsorptions for Pb(II)^☆^

**DOI:** 10.1016/j.heliyon.2024.e32422

**Published:** 2024-06-04

**Authors:** Jingyi Chen, Qianqian Duan, Chunyu Ji, Junsheng Liu, Ziyao Wang, Jiahui Song, Wei Li, Chaojian Zhang

**Affiliations:** a School of Energy, Materials and Chemical Engineering, Hefei University, 99 Jinxiu Avenue, Hefei, 230601, China; b State Key Laboratory of Materials-Oriented Chemical Engineering, Nanjing Tech University, Nanjing, 210009, China

**Keywords:** Modification, Biochar, Adsorption, Pb(II), Coconut shell, Mn-modified

## Abstract

The modified coconut shell biochars (MCSBCs) were fabricated and their adsorptions for Pb(II) were evaluated, in which waste coconut shell was used as the raw material, both ZnCl_2_ and KMnO_4_ were applied as the inorganic modifiers. FT-IR spectra, TGA, SEM and BET techniques were utilized to characterize their properties. It was spotted that the thermal stability of UCSBC could arrive at 500 °C. The BET specific surface areas of both Zn- and Mn-modified MCSBCs (485.137, 476.734 m^2^/g) were highly decreased as compared with that of UCSBC (3528.78 m^2^/g). In contrast, the average pore diameters of both Zn- and Mn-modified MCSBCs (3.295, 3.803 nm) were smaller than that of UCSBC (3.814 nm). These findings reveal that the modification of CSBC didn't change its pore size. Their adsorptions for Pb(II) were performed and some controlling factors involving pH, contact time, starting concentration and temperature were explored. Moreover, the experiment data were fitted via linear and non-linear techniques. It was found that the Langmuir maximal adsorption amounts of un-modified coconut shell biochar (UCSBC), Zn-modified and Mn-modified MCSBCs for Pb(II) could reach 31.653, 86.547 and 93.666 mg/g, respectively. Two-parameter kinetic models exposed that Pb(II) adsorption on UCSBC, Zn-modified and Mn-modified MCSBCs obeyed both the Lagergren first-order (non-linear R^2^ = 0.990, 0.954, 0.953, respectively) and Avrami fractional-order (non-linear R^2^ = 0.989, 0.946, 0.945, respectively) kinetic models. Two-parameter and three-parameter isotherm models verified that Pb(II) adsorption on UCSBC, Zn-modified and Mn-modified MCSBCs followed the Langmuir (non-linear R^2^ = 0.992, 0.997, 0.993, respectively) as well as Sips (non-linear R^2^ = 0.992, 0.997, 0.992, respectively) isotherm models. The computation of thermodynamic parameters evidenced that the modification of UCSBC via KMnO_4_ and ZnCl_2_ can effectively rise its adsorption for Pb(II), exhibiting promising applications in the handling of metal-bearing water.

## Highlights

• Modified coconut shell biochars were prepared using ZnCl_2_ and KMnO_4_ as modifiers.
• Mn-modified MCSBCs adsorptions for Pb(II) had the maximum q_e_ value of 93.67 mg/g.
• Both two- and three-parameter isotherm models were used to fit the testing data.
• MCSBCs showed excellent adsorption properties for Pb(II) from water.

## 1 Introduction

With the quick growth of industrialization around the world, the quantity of various industrial wastewaters was elevated to a new degree, these wastewaters contain different types of heavy metal ions, such as lead (Pb), copper (Cu), nickel (Ni), and cobalt (Co), Hg(II), etc. [[1], [2], [3], [4]]. Discharging these heavy metal ions into water source or water environment will not only pollute the ecological environment, but also may hurt the human body. Thus, when the level of heavy metal ions in human body reaches to a certain content, these substances will damage human's organs and cause acute or chronic poisoning and impairing [5]. Therefore, how to remove these toxic metal ions from wastewater has fascinated widespread eyes.

**Fig. 1:**
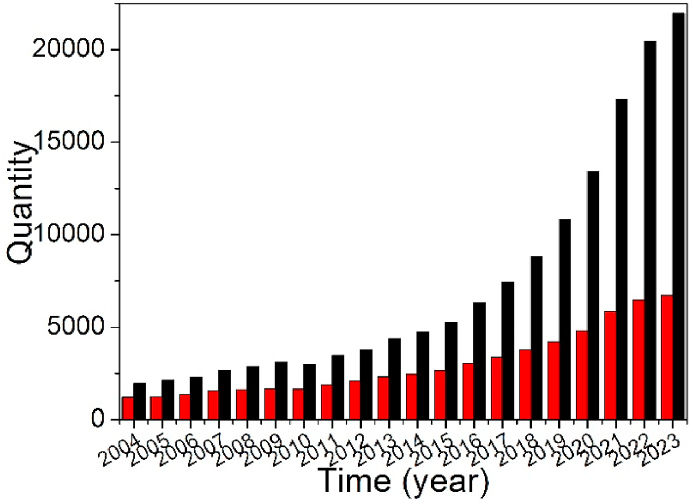
Chronology of publications on the study of heavy metal pollution and lead (Pb) contamination in the past 20 years indexed in ScienceDirect. Source: ScienceDirect database, https://www.sciencedirect.com. Advanced Search: Find articles with these terms: ‘heavy metal pollution (black color) or ‘lead contamination or Pb contamination’ (red color), Year(s): 2004–2023. Search date: March 28, 2024. (For interpretation of the references to color in this figure legend, the reader is referred to the Web version of this article.)

If we search ScienceDirect database, it can be seen that the chronology of publications on the study of both heavy metal pollution and lead (Pb) contamination all increases with the elapsed time in the past 20 years and increased highly since 2019 (as shown in Fig. 1). These data prove that the disposal of heavy metal pollution including lead becomes a hot topic and is an urgent issue.

For such a purpose, numerous efficient ways have been newly developed, which mainly include chemical precipitation [6], membrane separation [7,8], adsorption [[9], [10], [11]], electrodialysis [12], and so on. Among them, adsorption evidences such advantages as simple operation process, high cost-effectiveness, lost-cost and good removal effect [[10], [11], [12], [13], [14], [15]], especially for the treatment of dilute solution, the advantages of adsorption will be dominant. Taking adsorption technique into consideration, the choosing of fabrication materials are the key issue, which will determine the wide utilization of adsorbents in industry. Among many adsorbent materials, live carbon materials have frank advantages, which are characterized by wide sources, green and low cost [16,17]. Consequently, green low-carbon materials captures much eyes. Among which, waste coconut shell is one of important agricultural wastes in China, it can be processed to make coconut shell activated carbon and be widely used as a new type of porous biochar (BC) adsorbents in the process of carbonization of coconut shell carbon [18]. Typically, the outer surface of coconut shell is black and granular, with well-developed pores, stronger adsorption nature, higher strength, easy to be regenerated, economic and durable, and the prospect of making activated carbon from waste coconut shell for low/dilute concentration heavy metal wastewater treatment is broad [[18], [19], [20]]. Therefore, we establish a hypothesis that by functionalization modification of ordinary coconut shell carbon to fabricate a green low-cost bio-adsorbent, it can not only make it homogeneous disperse in aqueous solution, but also improve its specificity for heavy metal ions adsorption, thus its adsorption performance will be highly improved and lost-cost merits will be presented.

To create new type of modified biochars using waste coconut shell as a low-carbon material and rise their adsorption properties, herein, both KMnO_4_ and ZnCl_2_ modified coconut shell biochars (MCSBCs) were prepared as low-cost adsorbents, as a typical heavy metal ions, Pb(II) was selected as the target heavy-metal pollutant, thus the sorption behaviors of MCSBCs for Pb(II) will be explored. For comparison, the un-modified coconut shell biochar (UCSBC) was also be checked. Besides, constructed on different parameters theoretical equations, the adsorption kinetic and isotherm models will also be surveyed. The innovation of this research was the modification method and the improvement of adsorption efficiency. It is expected that the preparation cost of green adsorbents can be highly decreased and will make contributions to reduction of carbon dioxide emissions.

## 2 Experimental

### 2.1 Materials

The activated coconut shell carbon came from Henan Baidu Water Purification Materials Co., Ltd; KMnO_4_ (purity ≥99.5 %) came from Shanghai Jutai Special Reagent Co., Ltd; ZnCl_2_ (purity ≥98 %) came from Yantai Shuangshuang Chemical Co. All the reagents were analytical pure and used without further purification.

### 2.2 Preparation of modified CSBC

#### 2.2.1 Preparation of UCSBC

The commercially available large particles of coconut shell were broken using a 200G solid pulverizer and passed through a standard sieve of 100 mesh and 200 mesh to obtain the pristine coconut shell biochar (CSBC) of the desired particle size. Followed, the obtained CSBC was placed in a beaker and washed several times by adding an appropriate amount of tap water until the water was thoroughly clear and there was no suspended matter, and then it was put into a drying oven for drying at 105 °C; after the drying, the original CSBC was achieved. Subsequently, the original CSBC was placed in a muffle furnace to be roasted at 500 °C for 45 min, thus it was activated. After cooling to room temperature (25 °C), the activated CSBC was cleaned three times with tap water so as to remove the impurities produced during the process of roasting, and then it was dried at 105 °C thru a drying oven. Thus, the un-modified CSBC could be obtained and was named as UCSBC.

#### 2.2.2 Preparation of Zn-modified MCSBC

6 g of unroasted UCSBC was poured into 25 mL of 3 mol/L ZnCl_2_ solution and ultrasonicated for 1 h. After drying at 105 °C, it was placed in a muffle furnace and roasted at 450 °C for 2 h. After cooling to room temperature, it was washed with tap water to eliminate the impurities generated during the process of roasting, then it was dried at 105 °C through a drying oven to obtain ZnCl_2_-modified MCSBC and named as Zn-modified MCSBC.

#### 2.2.3 Preparation of Mn-modified MCSBC

**Fig. 2:**
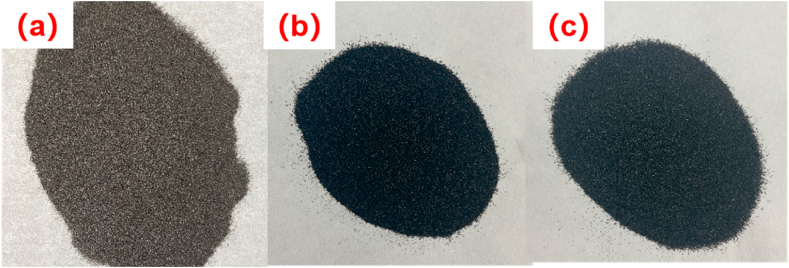
Appearance of UCSBC (a), Zn-modified (b) and Mn-modified (c) MCSBCs.

1g of unroasted CSBC was incorporated into 20 mL of 0.03 mol/L KMnO_4_ solution and placed in a magnetic stirrer to stir for about 4–5 h until the color of the solution faded, and then the modified activated carbon was washed and dried at 105 °C by water to obtain the KMnO_4_- modified MCSBC. It was named as Mn-modified MCSBC (Fig. 2 shows the appearance of the modified MCSBCs).

### 2.3 Characterizations

The thermal stability of MCSBCs was evaluated using a thermogravimetric analyzer (Q500, TA, USA), which limited the temperature range to about 25–800 °C under N_2_ protection.

The surface FE-SEM of MCSBCs was examined by a cold field emission scanning electron microscope (SU8010, Hitachi, Japan).

Infrared spectra of UCSBC and MCSBCs were analyzed by Fourier transform infrared spectrometer (Nicolet IS 50, Thermo Nicolet, USA).

The specific surface area and pore size of UCSBC and MCSBCs were determined using a fully automated specific surface area and porosimeter (Automatic-iQ, Quantachrome, USA).

### 2.4 Adsorption experiment

Toward investigate the adsorption of MCSBCs for Pb(II), batch experiment was done. The concentrations of Pb(II) were set as 20, 40, 60, 80, and 100 mg/L, individually. The pH was controlled at 2, 3, 4 and 5, and was adjusted with 1.0 mol/L HCl and NaOH. All the experiments were performed three times in parallel and the outcomes of the experiments were calculated as mean values. The sorption quantity of Pb(II) (q_t_, mg/g) on the UCSBC or MCSBCs can be acquired by Eq. (1).

(1) $$q_{t}=\frac{\left(C_{0}-C_{E}\right)V}{M}$$

In Eq. (1), C_0_ and C_E_ (mg/L) are the starting and remaining concentrations of Pb(II), individually; V (L) is the volume of solution; and M (g) is the mass of UCSBC or MCSBCs.

### 2.5 Regeneration cycle testing

To examine the regeneration and cycle times of UCSBC and MCSBCs, adsorption and de-sorption measurements were performed, in which HCl, HNO_3_ and EDTA (80 mL of 1.0 mol/L) were used as de-sorbents.

## 3 Results and discussion

### 3.1 FT-IR spectra

**Fig. 3:**
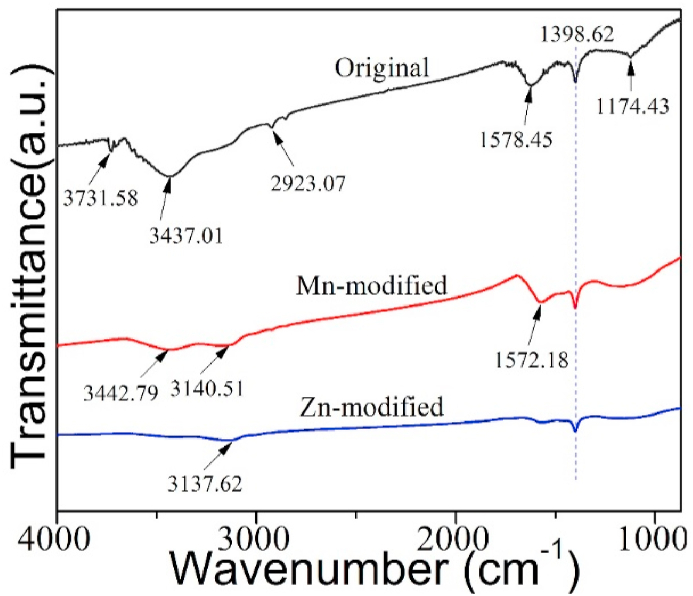
FT-IR profiles of UCSBC (black), Zn-modified (blue) and Mn-modified MCSBCs (red). (For interpretation of the references to color in this figure legend, the reader is referred to the Web version of this article.)

Toward probe the changes of functional groups on the modified MCSBCs, FT-IR spectra of the un-modified CSBC (i.e., original CSBC) and modified CSBC (i.e., MCSBCs) were performed and are presented in Fig. 3.

As displayed in Fig. 3, the spectra of UCSBC and MCSBCs have obvious transformation. For example, for the UCSBC, the characteristic vibration peaks appeared at near 3731, 3437, 2923, 1578, 1398 and 1174 cm^−1^. Among which, the vibration peaks appeared at 3731, and 3437 should be corresponded to the vibration region generated by –O-H or –NH_2_. While, the peak at 2923 cm^−1^ was assigned to –CH_2_. And the characteristic peak appeared at 1578 cm^−1^ should be ascribed to C=O of carboxylic groups in UCSBC [18]. The peak at neighboring 1398 cm^−1^ will be the vibration produced by –OH or the vibration from O–C single-bond skeleton of UCSBC.

In contrast, the position of peaks in MCSBCs was altered after modification by various metal modifiers. Taking Zn-modified MCSBC into account, in addition to the peak at 1398 cm^-^1 as witnessed in UCSBC, the peak position of –O-H was shifted to 3137 cm^−1^. While, the peak intensity of C=O at around 1578 cm^−1^ was highly weakened, suggesting that the quantity of C=O in ZnCl_2_-modified MCSBC possibly was reduced after the modification by ZnCl_2_.

As for Mn-modified MCSBC, besides the same peak as that of 1398 cm^−1^ with that from UCSBC, the characteristic peaks at 3442 and 3140 cm^−1^ linked toward the vibration region of –O-H, and the peak placed at 1572 cm^−1^ corresponded to the vibration created from C=O kept unchanged. This is because after the oxidative modification by KMnO_4_, the content of acidic oxygen-containing groups from its surface was elevated, which will uphold the complexation reaction of MCSBCs with Pb(II). It can be confirmed by the data obtained from the following adsorption experiment.

### 3.2 TGA curve of UCSBC

**Fig. 4:**
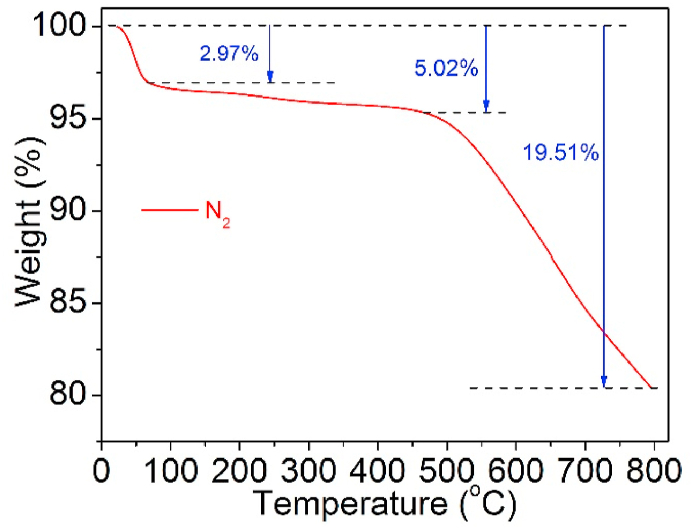
TGA curve of UCSBC.

To determine the pyrolysis temperature of the UCSBC, TGA measurement was performed. The TGA curve is presented in Fig. 4.

As shown in Fig. 4, three degradation steps are clear. The curve indicates a sharp drown trend before 100 °C, and the weight loss percentage of UCSBC is relatively large, the weight loss rate is around 2.97 % within this temperature scope. The reason should be credited to the volatilization of liquid in UCSBC. Moreover, the weight loss rate of UCSBC is obviously reduced in the temperature region of 100–500 °C, and the weight loss rate is near 5.02 %. Desorption of internally bound water and smaller molecular chains in UCSBC will be the main manner. As the temperature exceeds 500 °C, the weight loss rate (%) is ca. 19.51 %. The UCSBC starts to undergo a pyrolysis process during this step. the internal pores of the UCSBC will fully adsorb nitrogen and react with the volatile components left in the interior to a certain extent, during which the weight loss rate of the UCSBC increases compared with that of before, and the UCSBC will appear in the charring stage as the subsequent temperature increases, and the residual substances in the UCSBC will be slowly pyrolyzed until it was carbonized, and the remaining residue is basically carbide. Based on such a degradation process, the pyrolysis temperature of UCSBC and modified CSBC was chose as below 500 °C.

### 3.3 SEM images

**Fig. 5:**
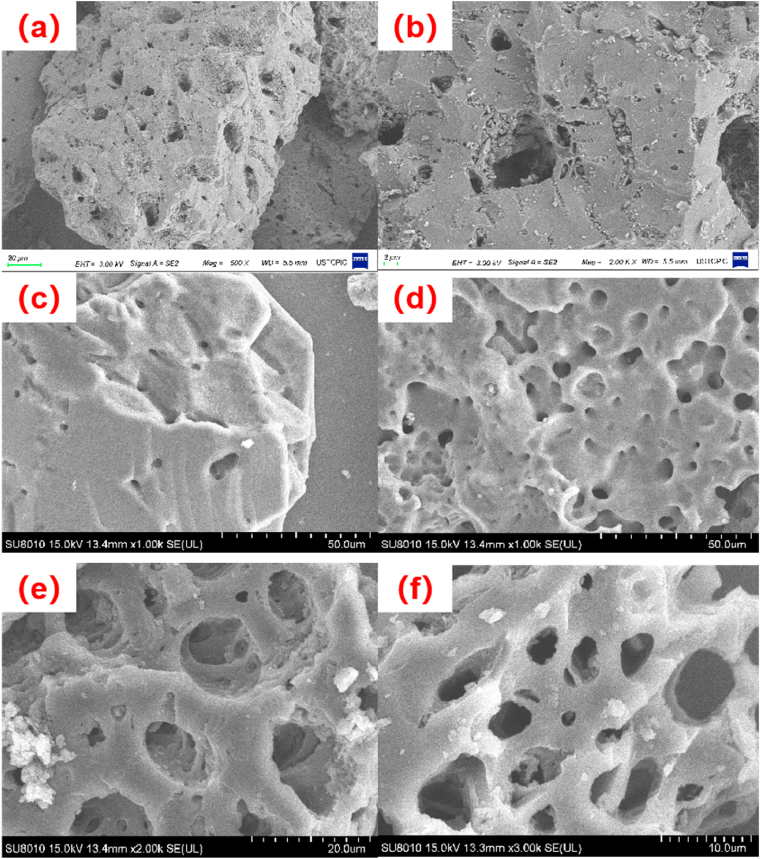
SEM images of UCSBC (a) 500x and (b) 2000x; Zn-modified MCSBC (c) 1000x and (d) 1000x, as well as Mn-modified MCSBC (e) 2000x and (f) 3000x.

For checking the changes in the surface structure of the UCSBC and MCSBCs, SEM mappings were observed and are shown in Fig. 5.

As disclosed in Fig. 5, the pore structure of UCSBC is clearly visible from the external morphology, and its surface is very smoother with the presence of a small number of natural UCSBC pores. In Fig. 5(c) and (d), the Zn-modified MCSBC contains more pores with different morphologies and sizes, the pore structure is richer than that of UCSBC, and the number of pores is improved compared with that of UCSBC, which may be due to the fact that ZnCl_2_ can combine with C on the active site of MCSBCs, which will strengthen the pore-making effect of UCSBC, and in turn, will make the number of its pores increase. From Fig. 5(e) and (f), it can be seen that the number of pores on the surface of Mn-modified MCSBC augmented, showing porous characteristics and fine particulate matter attached to the surface. The reason can be assigned to the fact that large number of acidic oxygen-containing functional groups were produced after the modification of CSBC by KMnO_4_, and the acidic groups increased with the increase in the amount of KMnO_4_, generating manganese dioxide and loaded on its surface.

### 3.4 BET measurement

**Fig. 6:**
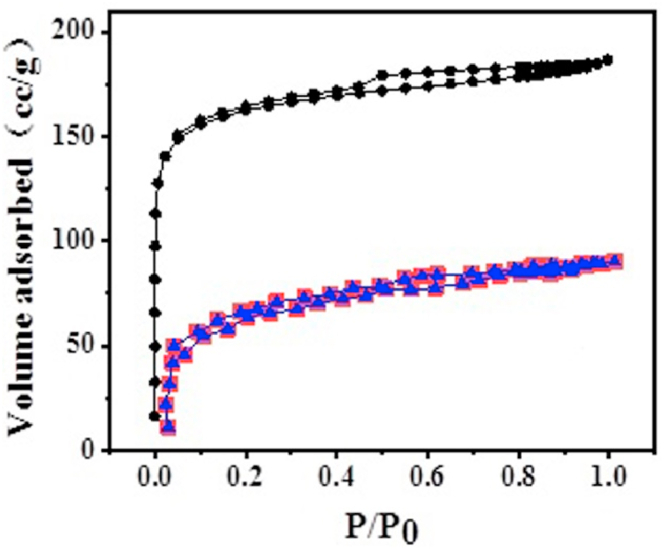
N_2_ adsorption/desorption isotherms of UCSBC(●), Zn-modified (■), and Mn-modified (▲) MCSBCs.

In order to determine the pore structure of UCSBC, Zn-modified and Mn-modified MCSBCs, BET tests were carried out and the data are displayed in Fig. 6. The specific surface area and pore size distribution parameters of UCSBC and MCSBCs are shown in Table 1.

**Table 1:** Specific surface area and pore size distribution of UCSBC, Zn-modified and Mn-modified MCSBCs.

Parameters	Biochar		
	UCSBC	Zn-modified	Mn-modified
**S**_**BET**_**(m**^**2**^**/g)**	3528.780	485.137	476.734
**S**_**mic**_**(m**^**2**^**/g)**	32.145	29.920	26.979
**S**_**mic**_**/S**_**BET**_**(%)**	0.912	6.170	5.660
**V**_**tot**_**(cc/g)**	0.289	0.287	0.140
**V**_**mic**_**(cc/g)**	0.038	0.037	0.039
**V**_**mic**_**/V**_**tot**_**(%)**	13.170	12.890	27.940
**D**_**P**_**(nm)**	3.814	3.295	3.803

As displayed in Fig. 6, The adsorption isotherms of Zn-modified and Mn-modified MCSBCs have the same changing trends, which are similar to that of UCSBC. While, the adsorption capacities of the three specimens increase to a limiting value near a straight line when the relative pressures (P/P_0_) are extremely low, which meets the requirements of the Type I adsorption isotherms [21], i.e., it is demonstrated that the adsorption isotherms of UCSBC, Zn-modified and Mn-modified MCSBCs all contain microporous structures and have no obvious hysteresis lines, representing that there are no large pores. The adsorption isotherm of UCSBC, Zn-modified and Mn-modified MCSBCs is located in the middle of the isotherm, such an adsorption isotherm is obviously not coincident with the detachment isotherm. That is, the detachment line is higher than the adsorption line, the three curves are all in good agreement with type I adsorption isotherm, and there is no hysteresis line in the curve under the low relative pressure. This disclosed that the adsorption curves at low P/P_0_ increased rapidly, while at high P/P_0_ the adsorption curves mainly showed microporous filling, and multilayer adsorption phenomenon may be occurred with the increase of P/P_0_, and capillary coalescence occurred at high P/P_0_, which exposed that the three kinds of coconut shell carbon contained mesoporous structure. It indicates that Mn-modified MCSBC have microporous-medium pore structure. Generally, micropore structure will provide most of the adsorption sites for heavy metal ions adsorbed by coconut shell carbon, while mesopore structure will determine the adsorption rate, which can not only be a channel for adsorbent particles to move to the interior of the micropores, but also directly adsorb certain substances with a slightly larger particle size that cannot be adsorbed by the micropores. Therefore, adsorbents with a mixed microporous-mesoporous structure are a better indication of adsorption effectiveness.

As shown in Table 1, the specific surface area of MCSBC was highly decreased after the modification by ZnCl_2_ and KMnO_4_. Interestingly, the specific surface areas of Zn-modified and Mn-modified MCSBCs (485.137, 476.734 m^2^/g) are all smaller than that of UCSBC (3528.78 m^2^/g), But, the ratios (**S**_**mic**_**/S**_**BET**_**, %**) of specific surface areas of micro-pores to the total specific surface area of Zn-modified and Mn-modified MCSBCs (6.17 %, 5.66 %, respectively) are larger than that of UCSBC (0.912 %). These data indicate that the microporous structure of CSBC modified by ZnCl_2_ and KMnO_4_ was highly improved and the modification by ZnCl_2_ and KMnO_4_ would decrease the specific surface areas of UCSBC. Nevertheless, by comparison of the average pore diameter and microporous volume of MCSBCs with UCSBC, they had little change, i.e., the average pore diameters of Zn-modified and Mn-modified MCSBCs (3.295, 3.803 nm, respectively) were smaller than that of UCSBC (3.814 nm). While, their microporous pore volumes (0.038, 0.037, and 0.039 cc/g) almost unchanged before and after modification. Among them, the volume ratio (V_mic_/V_tot_, %) of the microporous to the total pore of Mn-modified MCSBC (27.94 %) evidences the highest one, i.e., Mn-modified MCSBC had largest percentage of V_mic_/V_tot_. Such a micro-porous structure of Mn-modified MCSBC will be conducive to Pb(II) adsorption. Hence, from the view of adsorption of heavy metal ions, Mn-modified MCSBC expects to be the most effective biochar in attaching Pb(II).

From the BET data, it can be specified that the modification of CSBC by ZnCl_2_ and KMnO_4_ will be contributed to the generation of microporous structure in CSBC, while the enhancement of microporous structure in CSBC was mainly due to the reduction of mesoporous structure. The possible reason is that the reaction between ZnCl_2_ or KMnO_4_ with CSBC will result in the collapse and blockage of the original pore channels during the impregnation of CSBC, which made the UCSBC become rougher, and the mesopores collapsed into a number of micropores, and the UCSBC with a well-developed microporous structure is more favorable for the sorption of Pb(II), which can be confirmed by is the results of the following experiments.

### 3.5 Adsorption of UCSBC and MCSBCs for Pb(II)

#### 3.5.1 Effect of solution pH

**Fig. 7:**
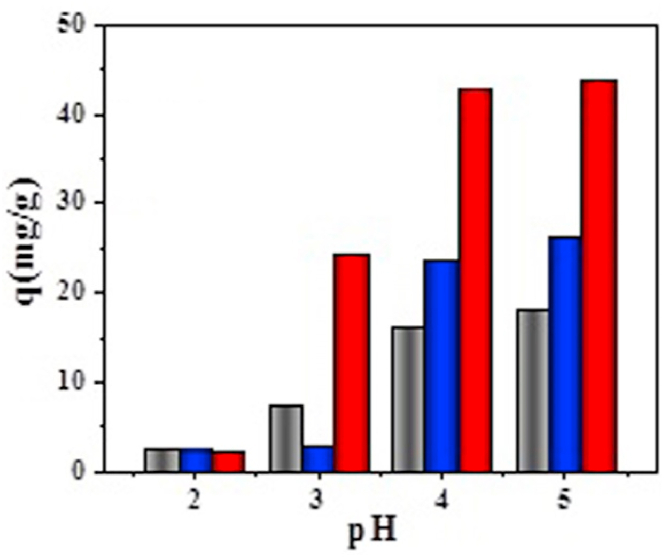
Effect of pH on UCSBC (black), Zn-modified (blue) and Mn-modified MCSBCs (red) for Pb(II) adsorption, respectively. Conditions: the concentration of Pb(II) = 80 mg/L,V = 80 mL, and dosage = 0.1g for 24 h at 25 °C. (For interpretation of the references to color in this figure legend, the reader is referred to the Web version of this article.)

Generally, the pH of solution will largely impact the adsorption of Pb(II) in aqueous solution. As reported [22,23] that Pb(II) will be precipitated as the pH was higher than 5.5. Similar finding can be found in an article, in which it was reported [24] that lead cations begin to precipitate at pH 5.2. Thus the pH values of solution in this case were set within the region of 2–5 and the graphs are depicted in Fig. 7.

From Fig. 7, it is clear that pH values had significant influences on Pb(II) sorption. For instance, the sorption amount of Pb(II) on the top of UCSBC and as well as on Zn-modified and Mn-modified MCSBCs was very low as the pH was equal to 2. This is because the lower the pH, the greater the H^+^ concentration, which will make more H^+^ compete with Pb(II) due to the repulsion effect between H^+^ and Pb(II) (i.e., H^+^ ↔ Pb^2+^). Alternatively, the lower H^+^ concentration will result in the protonation of ionic groups and sheltered the active sites onto the surface of them. As a result, less Pb(II) can be adsorbed. Differently, the sorption amount of Pb(II) was improved with an increase in solution pH and reached the largest quantity at pH 5. In contrast, as the pH was larger than 5, the amount of OH^−^ will be increased. Thus, OH^−^ will combine with Pb^2+^ to produce the products such as Pb(OH)^+^ or Pb(OH)_2_ [i.e., OH^−^ + Pb^2+^ ↔ Pb(OH)^+^ or 2OH^−^ + Pb^2+^ ↔ Pb(OH)_2_]. These species will hinder the adsorption of Pb(II) on the surface of UCSBC and MCSBCs. Accordingly, the sorption quantity of Pb(II) on them will be highly reduced.

In addition, Pb(II) sorption onto the three kinds of CSBC indicated different trends within the pH region of 2–5. Among which, Mn-modified MCSBC had the best adsorption effect on Pb(II). This is because the modification of CSBC by KMnO_4_ will generate the neo-eco-MnO_2_, and at the same time, the oxygen-containing groups onto the surface of Mn-modified MCSBC were increased, which made its adsorption effect be enhanced. In contrast, after modification by ZnCl_2_, its adsorption effect was enhanced as pH increased compared with UCSBC, this is because ZnCl_2_ could combine with C on the active sites, which strengthened the pore-making effect on Zn-modified MCSBC and formed rich graphite microcrystalline structure. Accordingly, its adsorption effect for Pb(II) was enhanced.

#### 3.5.2 Effect of contact time

**Fig. 8:**
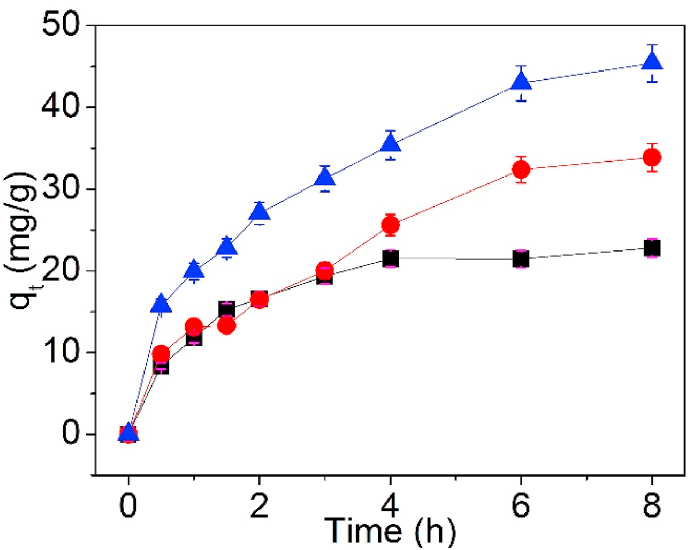
Effect of time on UCSBC (■), Zn-modified (●) and Mn-modified (▲) MCSBCs for Pb(II), conditions: the concentration of Pb(II) = 80 mg/L, V = 80 mL, and dosage = 0.1g at 25 °C.

For understanding the effect of contact time onto Pb(II) sorption, the relationship of adsorption amount of Pb(II) against contact time was measured and is portrayed in Fig. 8.

From Fig. 8, the adsorption amount of UCSBC, Zn-modified and Mn-modified MCSBCs for Pb(II) presented the same changing trends: all increased with the prolong of contact time. However, their adsorption amounts were different. Among them, Mn-modified MCSBC had the largest adsorption quantity for Pb(II), demonstrating that the modification of CSBC by KMnO_4_ will be conducive to the sorption of Pb(II) on MCSBC. Meanwhile, the sorption rate of them was very fast in the first 4 h. With the elapse of the contact time, the sorption rate was slowed down and tended to arrive at an equilibrium state. Clearly, the Pb(II) adsorption reached a stabilization after adsorption for about 8 h. The theoretical interpretation to these trends can be seen as follows.

In the first 4h, the surfaces of UCSBC, Zn-modified and Mn-modified MCSBCs contain abundant adsorption active sites, which can rapidly combine with Pb(II) in a relatively short time, so the change of adsorption amount in the first 4 h is obvious. Further, with the proceeding of Pb(II) adsorption, the active sites was occupied and the amount of sorption active spots on the surface of BC is reduced gradually and arrived at saturation state. Thus the adsorption will approach an equilibrium state. Accordingly, the sorption amount of Pb(II) will keep a stable state.

#### 3.5.3 Effect of starting concentration

**Fig. 9:**
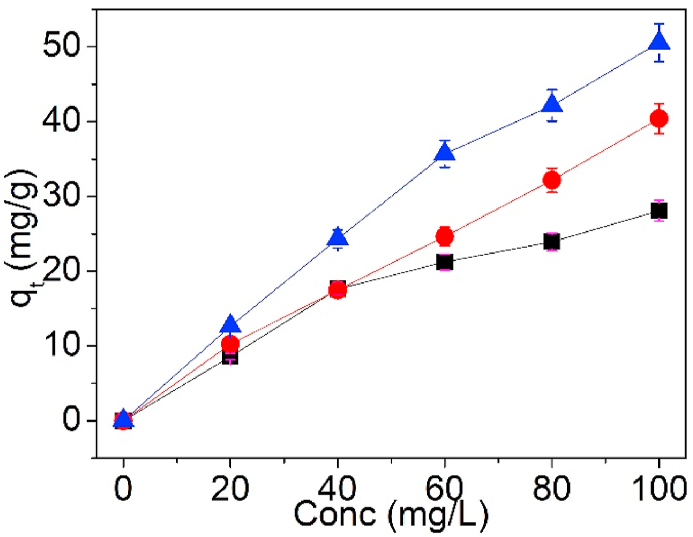
Effect of starting concentration on UCSBC (■), Zn-modified (●) and Mn-modified (▲) MCSBCs for Pb(II), conditions: contact time 24 h, pH = 5, and dosage = 0.1g at 25 °C.

Toward insight into the effect of starting concentration of solution on Pb(II) sorption on UCSBC, Zn-modified and Mn-modified MCSBCs, batch experiments were done, in which the starting concentration was changed from 20, 40, 60, 80, to 100 mg/L. Fig. 9 reveals the variation of starting concentration of solution on the sorption capacity of Pb(II) on UCSBC, Zn-modified and Mn-modified MCSBCs.

As seen in Fig. 9, for UCSBC, Zn-modified and Mn-modified MCSBCs, the sorption capacity of Pb(II) all raised with the increasing starting concentration of solution. This is because the more concentration of heavy metal ions are presented in the solution, the more individual metal ions are averaged to each active site on the CSBC, leading to a rise in the sorption amount of Pb(II) with the increasing concentration. Therefore, the above phenomena are reasonable and meet the expectation of experiment. Moreover, if the individual sample was taken into account, the changing trend was various. Among them, Mn-modified MCSBC had the highest adsorption amount of Pb(II). This outcome evidences that the modification of CSBC by KMnO_4_ will create more active sites on its surface due to the stronger oxidation of KMnO_4_. Thus the surface functional groups of MCSBCs will be highly boosted and the active sites are according increased. As a result, its adsorption nature for Pb(II) will be raised. Moreover, the larger percentage of V_mic_/V_tot_ of Mn-modified MCSBC will also be helpful for the adsorption of Pb(II) on its surface. The qe value of Pb(II) on Mn-modified MCSBC will thus be elevated.

#### 3.5.4 Effect of solution temperature

**Fig. 10:**
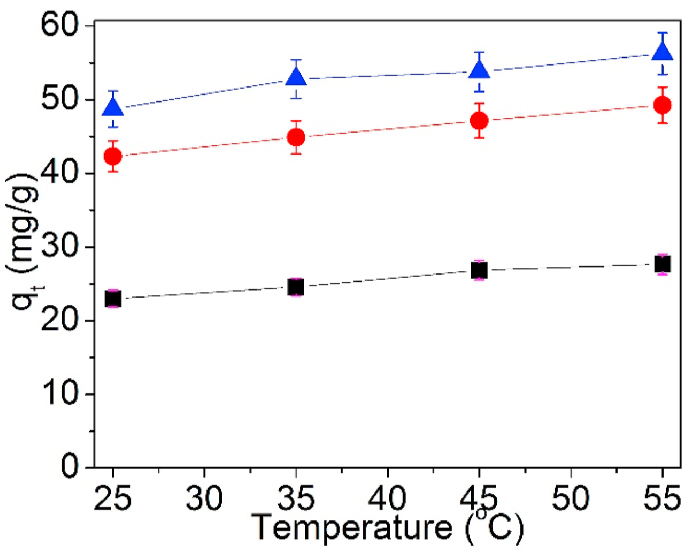
Effects of adsorption temperature on UCSBC (■), Zn-modified (●) and Mn-modified (▲) MCSBCs for Pb(II), conditions: the concentration of Pb(II) was 80 mg/L, and dosage = 0.1g for 24 h at pH 5.

Toward explore the effect of solution temperature onto Pb(II) adsorption on UCSBC and MCSBCs, adsorption experiment in the temperature scope of 25, 35, 45 and 55 °C was performed. Fig. 10 displays the function of solution temperature against adsorption amount of Pb(II).

As evinced in Fig. 10, the adsorption amount of Pb(II) on UCSBC, Zn- and Mn-modified MCSBCs all increased with the increasing temperature, signifying that Pb(II) sorption on these BCs was a endothermic process, it can be further confirmed via the calculation data of thermodynamic parameters (cf. Table 6). Moreover, for the individual sample, the adsorption amount of Pb(II) on UCSBC, Zn-modified and Mn-modified MCSBCs evidenced such an order as: UCSBC < Zn-modified < Mn-modified MCSBCs, i.e., Mn-modified MCSBC had the largest adsorption amount of Pb(II) among them. While, the q values of Pb(II) on Mn-modified MCSBC increased as the solution temperature was raised. This result further verified that the modification of CSBC by KMnO_4_ will be advantageous to Pb(II) adsorption.

#### 3.5.5 Regeneration cycle measurement

**Fig. 11:**
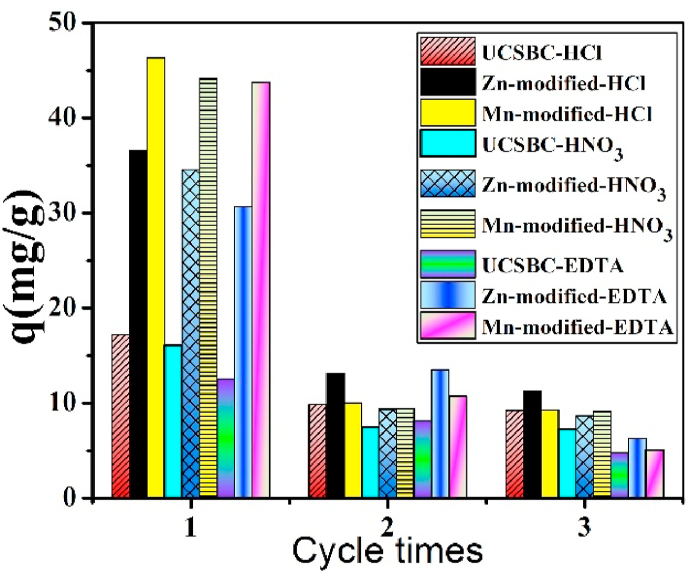
Regeneration cycle times of UCSBC and MCSBCs for Pb(II), conditions: V = 80 mL, the concentration of Pb(II) was 80 mg/L, and dosage = 0.1g for 24 h at 25 °C.

It is well accepted that for an industrial application, regeneration cycle of an adsorbent are significantly important. To insight into the cycle times of UCSBC, Zn- and Mn-modified MCSBCs, adsorption and desorption testing were measured, in which HCl, HNO_3_ and EDTA were used as de-sorbents. The dependency of q values against cycle times is exhibited in Fig. 11 and the relevant data are tabulated in Table 2.

**Table 2:** The adsorption amount of cycle times and reduction percentage.

Cycle times	De-sorbent								
	HCl			HNO_3_			EDTA		
	UCSBC	Zn- modified	Mn-modified	UCSBC	Zn- modified	Mn-modified	UCSBC	Zn- modified	Mn-modified
1	17.195	36.56	46.325	16.077	34.549	44.141	12.533	30.648	43.720
2	9.860	13.128	10.024	7.483	9.333	9.435	8.133	13.496	10.720
3	9.228	11.243	9.272	7.253	8.629	9.112	4.752	6.256	5.064
Reduction (%) of 2nd/1st	46.33	69.25	79.98	54.89	75.02	79.36	62.08	79.59	88.42
Reduction (%) of 3rd/2nd	6.41	14.36	7.50	3.07	7.54	3.42	41.57	53.65	52.76

In Fig. 11, the desorption quantities of UCSBC, Zn- and Mn-modified MCSBCs for Pb(II) all declined with the increasing cycle times. But, for the individual sample, its desorption degree and reduction percentage are different (see Table 2). Especially, these data using HCl as a de-sorbent were lower than those using HNO_3_ and EDTA as de-sorbents. For example, for UCSBC, the cycle number using HCl as a de-sorbent decreased from 17.195 to 9.86, 9.228 mg/g, i.e., around 46.33 % reduction after second cycle and 6.41 % reduction after third cycle. The reduction degree using HNO_3_ and EDTA as de-sorbents decreased around 54.89, 62.08 % reduction after second cycle and 3.07, 41.57 % reduction after third cycle, respectively. On the contrary, for Zn- and Mn-modified MCSBCs, their desorption degrees and reduction percentages are all larger than that of UCSBC. Considering the de-sorption degree and reduction percentage, it is apparent that HNO_3_ is an efficient de-sorbent for the regeneration of UCSBC, Zn- and Mn-modified MCSBCs.

Notice that, the regeneration cycle times of UCSBC, Zn- and Mn-modified MCSBCs are quite minor. Considering its low cost of waste coconut shell and other economic factors, such a regeneration step can be neglected in industry.

### 3.6 Kinetic model

Toward survey the correctness of experimental data, the matching of experimental data with some theoretical equations is considered as an effective tool to evaluate its matching degree. As one of typical theoretical equations, kinetic model can not only explore the sorption type of metal ions onto an adsorbing material, but also calculate the equilibrium sorption amount of metal ions on this adsorbent. The establishment of kinetic models are mainly dependent on the relationship of sorption amount of adsorbents for metal ions versus contact time.

Presently, two-parameter kinetic models are primarily included Lagergren first-order (1st-O) and second-order (2nd-O) kinetic equations [[24], [25], [26]], intra-particle diffusion (Ip-D) model [27,28], Elovich model [29,30], diffusion-chemisorption (Di-Ch) model [31,32], and Avrami fractional-order model [33,34]. Unfortunately, three-parameter kinetic model is unavailable so far. Thus, the sorption data of UCSBC, Zn- and Mn-modified MCSBCs for Pb(II) will be molded by these two-parameter kinetic models.

#### 3.6.1 Lagergren kinetic model

Both 1st-O and 2nd-O kinetic equations [25,26] are promising tool to discover the sorption activities of metal ions on an adsorbent. They usually are expressed as linear or non-linear Eqs. (2a), (2b), (3a), (3b), respectively.

(2a) $$q_{t}=q_{e}-q_{e}e^{-k_{L1}t}$$

Or

(2b) $$\ln\left(q_{e}-q_{t}\right)=-k_{L1}t+\ln\left(q_{e}\right)$$

(3a) $$q_{t}=\frac{q_{e}^{2}k_{L2}t}{\left(1+q_{e}k_{L2}t\right)}$$

Or

(3b) $$\frac{t}{q_{t}}=\left(\frac{1}{q_{e}}\right)t+\frac{1}{k_{L2}q_{e}^{2}}$$

Where k_L1_ (1/h) and k_L2_ (g/h·mg) stand for the rate constants of equations, respectively. q_t_ and q_e_ (mg/g) denote the adsorption amount of metal ions at time t and steadiness state, singly.

**Fig. 12:**
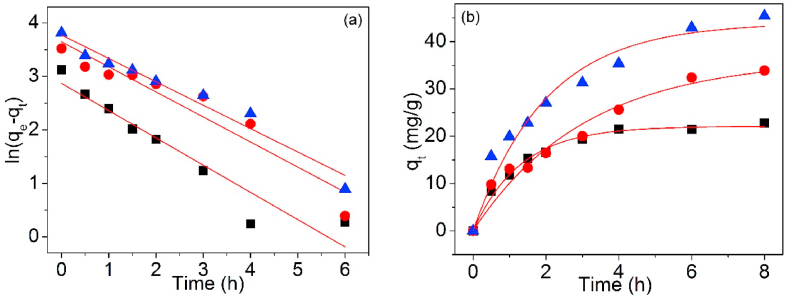
1st-O kinetic model of UCSBC (■), Zn-modified (●) and Mn-modified (▲) MCSBCs for Pb(II) adsorption, (a) linear fitting, (b) non-linear fitting.

**Fig. 13:**
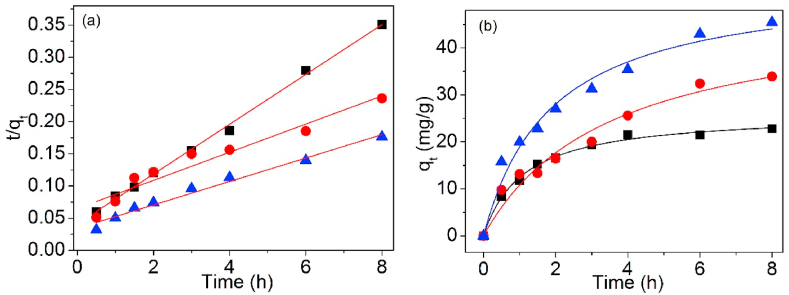
2nd-O kinetic model of UCSBC (■), Zn-modified (●) and Mn-modified (▲) MCSBCs for Pb(II) adsorption, (a) linear fitting, (b) non-linear fitting.

Fig. 12, Fig. 13 are the linear and non-linear fitting curves of Pb(II) adsorption on UCSBC, Zn-modified and Mn-modified MCSBCs, singly. The model factors are shown in Table 3.

**Table 3:** Adsorption kinetic parameters of linear and non-linear fitting.

Model	Fitting	Parameter	Biochar		
			UCSBC	Zn-modified	Mn-modified
1st-O	Linear	k_1_ (h^−1^)	0.509	0.468	0.437
		q_cal_ (mg/g)	17.654	38.421	43.555
		R^2^	0.904	0.899	0.954
		χ^2^	1.156	0.608	0.0749
	Non-linear	k_1_ (h^−1^)	0.772	0.325	0.504
		q_cal_ (mg/g)	22.101	36.285	43.949
		R^2^	0.990	0.954	0.953
		χ^2^	0.0207	0.170	0.0463
2nd-O	Linear	k_2_ (g/mg⋅h)	0.0366	0.00737	0.00973
		ISR^a^	24.420	15.396	29.315
		q_cal_ (mg/g)	25.814	45.709	54.895
		q_exp_ (mg/g)	22.788	33.882	45.399
		R^2^	0.997	0.922	0.979
		χ^2^	0.401	4.128	1.986
	Non-linear	k_2_ (g/mg⋅h)	0.0346	0.00586	0.00979
		q_cal_ (mg/g)	26.151	48.666	54.376
		R^2^	0.994	0.962	0.975
		χ^2^	0.496	6.450	1.775
Ip-D	Linear	K_Ip_ (mg/g· h^1/2^)	8.078	12.368	15.990
		x_Ip_ (mg/g)	3.250	−0.0599	2.998
		R^2^	0.893	0.982	0.983
Elovich	Linear	a_El_ (mg/g)	10.113	10.195	16.526
		b_El_ (mg/g)	6.821	10.780	13.578
		R^2^	0.659	0.785	0.738
Di-Ch	Linear	K_DC_ (mg/g)/min^0.5^	16.356	12.579	23.355
		q_cal_ (mg/g)	50.172	974.713	148.867
		R^2^	0.799	−0.152	0.869
Avrami fractional-order	Linear	kav	0.734	0.394	0.490
		n	0.798	0.828	0.734
		R^2^	0.972	0.846	0.923
	Non-linear	qe	22.101	36.284	43.949
		kav	0.878	0.570	0.710
		n	0.878	0.570	0.710
		R^2^	0.989	0.946	0.945
		χ^2^	0.0207	0.170	0.0463

a Initial sorption rate (ISR) = k_2_*q_e_^2^.

To examine the goodness of curve fitting of these theoretical models, chi-square (χ^2^) is considered as an excellent judging technique [35,36]. The χ2 data of 1st-O and 2nd-O kinetic equations were also programmed in Tables 3 and in which χ^2^ can be acquired using Eq. (4).

(4) $$\chi^{2}=\frac{\sum_{i=1}^{n}{\left(q_{e,cal}-q_{e,\exp}\right)}_{i}^{2}}{q_{e,\exp}}$$

As disclosed in Table 3, compared the values of correlation coefficients R^2^ of 1st-O with 2nd-O kinetic models for Pb(II) adsorption, the R^2^ values of 2nd-O kinetic model are all higher than those of 1st-O one no matter what the linear and non-linear fittings. However, if the χ^2^ values were considered, the χ^2^ values of 1st-O kinetic model were all lower than those of 2nd-O kinetic model and these data were also quite minor. Based on these information, it can be established that Pb(II) adsorption on UCSBC, Zn-modified and Mn-modified MCSBCs followed well with 1st-O kinetic model, i.e., such an adsorption process belonged to a physical manner and ion exchange was the rate-controlling stage, rather than a chemisorption style [37,38]. This finding can be further ascertained by Elovich model as discussed below.

Furthermore, compared the non-linear fitting data of 1st-O kinetic model with the linear ones, it is obvious that the R^2^ values of non-linear fitting were higher than those of linear one. Meanwhile, the χ^2^ values of non-linear fitting were highly lesser than those of linear fitting. Hence, it can be deduced that non-linear fitting method was more precise than the linear one.

#### 3.6.2 Intra-particle diffusion (Ip-D) model

For probing the effect of Pb(II) adsorption whether from exterior functional groups or from the pores of CSBC, Pb(II) adsorption on UCSBC, Zn-modified and Mn-modified MCSBCs was analyzed by Ip-D model [27,28]. Its expression can be given in Eq. (5).

(5) $$q_{t}=k_{Ip}t^{1/2}+x_{Ip}$$

In Eq. (5), q_t_ (mg/g) signifies the adsorbed amount at time t; k_Ip_ (mg/g· h^1/2^) is the rate constant for the Ip-D model; and x_Ip_ (mg/g) is the intercept and relates to the wideness of boundary layer.

**Fig. 14:**
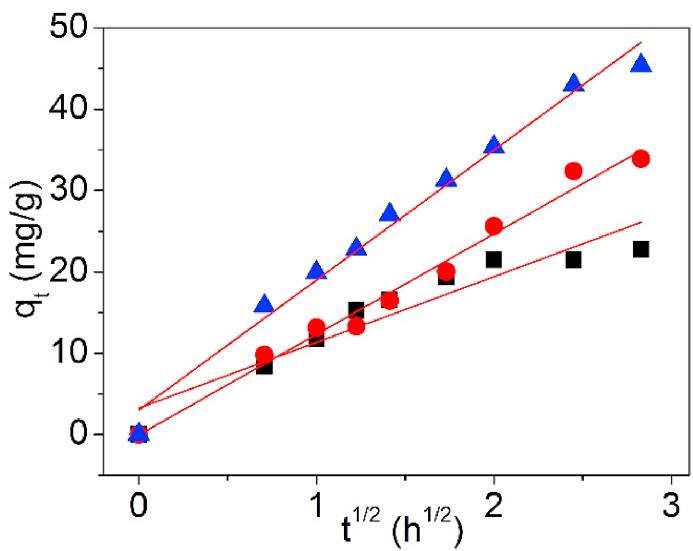
Intra-particle diffusion model of UCSBC (■), Zn-modified (●) and Mn-modified (▲) MCSBCs for Pb(II) adsorption.

Fig. 14 is the Ip-D model of UCSBC, Zn-modified and Mn-modified MCSBCs for Pb(II) adsorption. The model data are recorded in Table 3.

As in Table 3, the R^2^ data of UCSBC, Zn-modified and Mn-modified MCSBCs revealed high values. While, the intra-particle diffusion coefficient k_Ip_ of UCSBC, Zn-modified and Mn-modified MCSBCs increased from UCSBC, Zn-modified to Mn-modified MCSBC. Namely, among them, Mn-modified MCSBC had the largest intra-particle diffusion coefficient although it had the smallest specific surface area and total pore volume. The reason possibly is correlated to the ratio of V_mic_/V_tot_ (%), resulting in larger diffusion speed inside pores.

Furthermore, by comparison of the x_Ip_ values of UCSBC, Zn-modified and Mn-modified MCSBCs, it is obvious that the x_Ip_ value of Zn-modified MCSBC was almost close to the origin, suggesting that Pb(II) adsorption on Zn-modified MCSBC obeyed the intra-particle diffusion model, i.e., Pb(II) adsorption on Zn-modified MCSBC was chiefly administered by intra-particle diffusion. Conversely, the x_Ip_ values of UCSBC, and Mn-modified MCSBC were away from the origin, demonstrating that Pb(II) adsorption on them didn't followed the intra-particle diffusion model. Multiple diffusion effects will be the leading controlling manner for Pb(II) adsorption.

#### 3.6.3 Elovich model

Elovich model [29,30] can be exploited to describe the kinetic relations of chemisorption of various metal ions on the surface of adsorbents.

(6) $$q_{t}=a_{El}\;\ln\;t+b_{El}$$

In Eq. (6), a_El_ and b_El_ (mg/g) are the model coefficients.

**Fig. 15:**
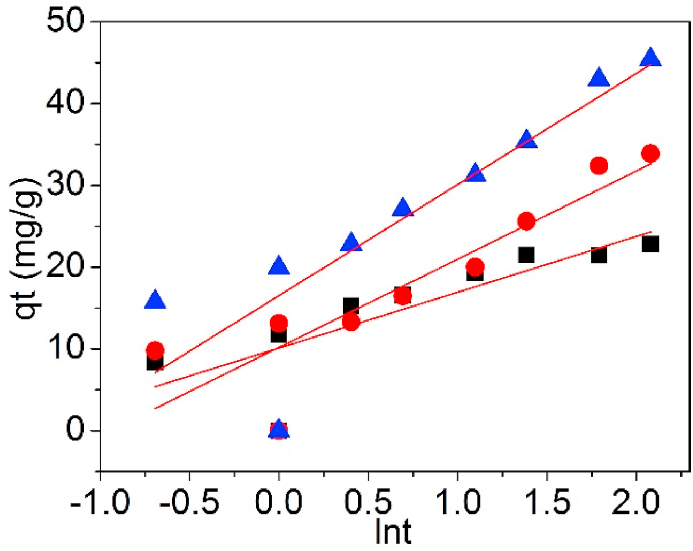
Elovich model of UCSBC (■), Zn-modified (●) and Mn-modified (▲) MCSBCs for Pb(II) adsorption.

Fig. 15 is the fitting curves of UCSBC, Zn-modified and Mn-modified MCSBCs based on Elovich model, and the data are detailed in Table 3.

As realized in Table 3, the R^2^ values of UCSBC, Zn-modified and Mn-modified MCSBCs were all much lower, signifying that Pb(II) adsorption on UCSBC, Zn-modified and Mn-modified MCSBCs didn't abided by Elovich model. Specifically, the adsorption activities of Pb(II) on UCSBC, Zn-modified and Mn-modified MCSBCs cannot be interpreted through Elovich model. Therefore, Pb(II) sorption on UCSBC, Zn-modified and Mn-modified MCSBCs is not merely controled via the chemisorption way. This finding is agreement with the conclusion obtained from the Lagergren 1st-O and 2nd-O kinetic equations as mentioned before.

#### 3.6.4 Diffusion-chemisorption (Di-Ch) model

Differentiating from Elovich model, Di-Ch model is usually employed to insight into the sorption of metal ions onto a heterogeneous surface [31,32].

(7) $$\frac{t^{1/2}}{q_{t}}=\frac{1}{q_{e}}\left(t^{1/2}\right)+\frac{1}{K_{D-ch}}$$

In Eq. (7), K_Di-Ch_ is the coefficient of Di-Ch model.

**Fig. 16:**
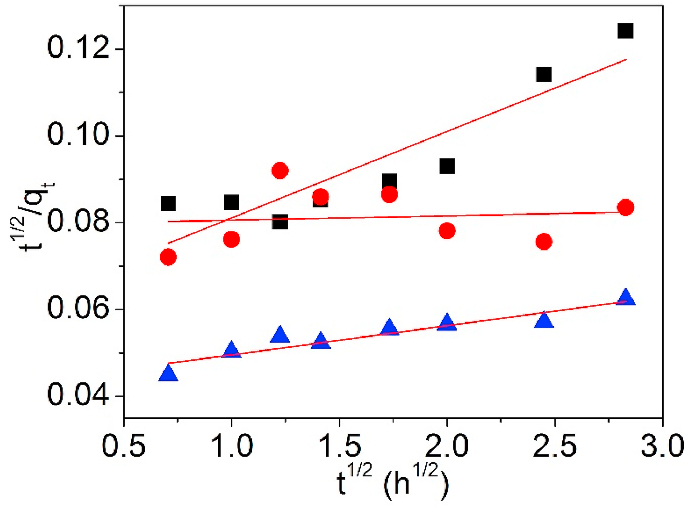
Di-Ch model of UCSBC (■), Zn-modified (●) and Mn-modified (▲) MCSBCs for Pb(II) adsorption.

Fig. 16 is the fitting curves of K_Di-Ch_ model and the pertinent data are detailed in Table 3.

As summarized in Table 3, the fitting grade of Pb(II) adsorption on UCSBC, Zn-modified and Mn-modified MCSBCs was very lower based on Di-Ch model. Consequently, Pb(II) adsorption on UCSBC, Zn-modified and Mn-modified MCSBCs didn't obeyed Di-Ch model. That is, adsorption process of Pb(II) on UCSBC, Zn-modified and Mn-modified MCSBCs was not governed the diffusion-chemisorption way.

#### 3.6.5 Avrami fractional-order model

Avrami fractional-order model [33,34] exposes the manifold kinetic courses. It is usually linked to the kinetic thermal decomposition and can be linearly or non-linearly written as Eq. (8a), (8b).

(8a) $$q_{t}=q_{e}\left[1-e^{-{\left(k_{avr}t\right)}^{n}}\right]$$

or,

(8b) $$\ln\left[-\ln\left(1-\frac{q_{t}}{q_{e}}\right)\right]=n\;\ln\;t+n\;\ln\;k_{avr}$$

In Eq. (8a), (8b), k_avr_ is the Avrami fractional-order rate constant, and n is the model constant.

**Fig. 17:**
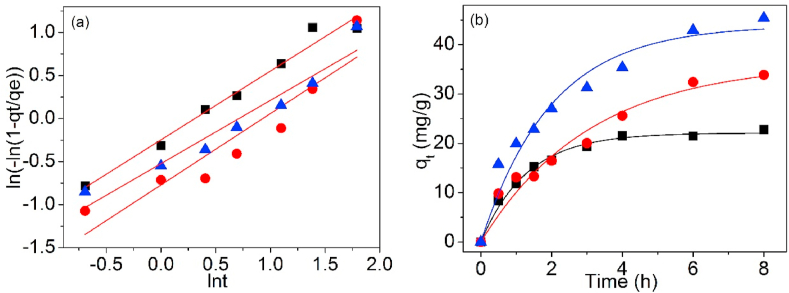
Avrami fractional-order model of UCSBC (■), Zn-modified (●) and Mn-modified (▲) MCSBCs for Pb(II) adsorption, (a) linear fitting, (b) non-linear fitting.

Fig. 17 is the fitting curves of Pb(II) adsorption on UCSBC, Zn-modified and Mn-modified MCSBCs via linear and non-linear method. The data are summarized in Table 3.

As evidenced in Table 3, the R^2^ data of linear fitting of UCSBC, Zn-modified and Mn-modified MCSBCs were all lower than those of non-linear ones, implying that non-linear fitting is more appropriate to define the adsorption performances of Pb(II) on UCSBC, Zn-modified and Mn-modified MCSBCs. Moreover, it is obvious that the R^2^ values of non-linear fitting of UCSBC, Zn-modified and Mn-modified MCSBCs were much higher (R^2^ values were closer to 0.95). Meanwhile, the χ^2^ values were much lower. These results demonstrated that Pb(II) adsorption on UCSBC, Zn-modified and Mn-modified MCSBCs can be interpreted by means of Avrami fractional-order model. Specifically, Pb(II) adsorption on UCSBC, Zn- and Mn-modified MCSBCs are manifold kinetic manners.

Note that, the R^2^ values of 1st-O kinetic model were all slightly larger than those of Avrami fractional-order model via non-linear fitting. Interestingly, these two models had almost the same q_cal_ and χ^2^ values. Additionally, the n values of Avrami fractional-order model for UCSBC, Zn-modified and Mn-modified MCSBCs were located within the scope of 0.57–0.88, which were larger than 0.5 and closer to 1. These information provide a clue that both 1st-O and Avrami fractional-order kinetic models all can be applied to interpret the adsorption activities of Pb(II) on UCSBC, Zn-modified and Mn-modified MCSBCs.

### 3.7 Isotherm model

For further exploring the sorption properties of Pb(II) on UCSBC, Zn-modified and Mn-modified MCSBCs, adsorption isotherm models were probed. Presently, there have two categories of adsorption isotherm models, i.e., two- and three-parameter isotherms. Specifically, two-parameter isotherms primarily enclose Langmuir, Freundlich, Temkin, and Dubinin–Radushkevich (D–R) isotherm models [[39], [40], [41], [42]]. Contrarily, three-parameter isotherm models generally encompass Sips, Redlich–Peterson (R–P), and Toth isotherms [[43], [44], [45]]. They will be discussed in detail as follows.

#### 3.7.1 Two-parameter isotherm model

##### 3.7.1.1 Langmuir isotherm model

Langmuir adsorption isotherm model [39,40] principally describes the adsorptive actions of metal ions occurred as the form of monolayer and is usually linearly or non-linearly expressed as Eq. (9a), (9b).

(9a) $$q_{e}=\frac{q_{m}K_{L}C_{e}}{1+K_{L}C_{e}}$$

Or

(9b) $$\frac{C_{e}}{q_{e}}=\left(\frac{1}{q_{m}}\right)C_{e}+\frac{1}{q_{m}K_{L}}$$

In Eq. (9a), (9b), q_e_ (mg/g) denotes the adsorption amount of metal ions at steadiness state; q_m_ (mg/g) is the maximal adsorption amount at saturation state; K_L_ means the Langmuir isothermal constant.

##### 3.7.1.2 Freundlich isotherm model

Freundlich adsorption isothermal model [39,40] generally defines the inhomogeneous sorption of metal ions on the surface of an adsorbents and can be linear or non-linear given as Eq. (10a), (10b).

(10a) $$q_{e}=k_{F}C_{e}^{\frac{1}{n}}$$

Or

(10b) $$\ln\left(q_{e}\right)=\frac{1}{n}\ln\left(C_{e}\right)+\ln\;k_{F}$$

In Eq. (10a), (10b), K_F_ is the Freundlich isothermal constant; and 1/n is an empirical parameter.

**Fig. 18:**
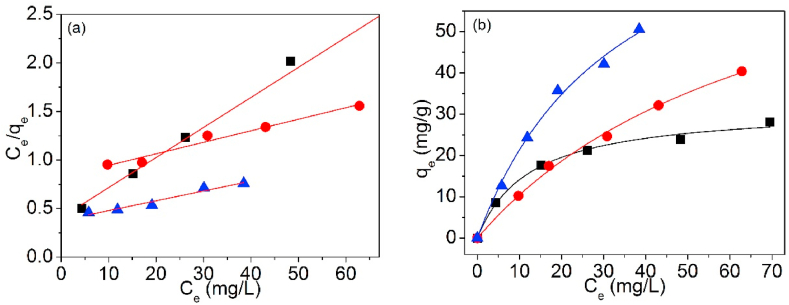
Langmuir isothermal model of UCSBC (■), Zn-modified (●) and Mn-modified (▲) MCSBCs for Pb(II) adsorption, (a) linear fitting, (b) non-linear fitting.

**Fig. 19:**
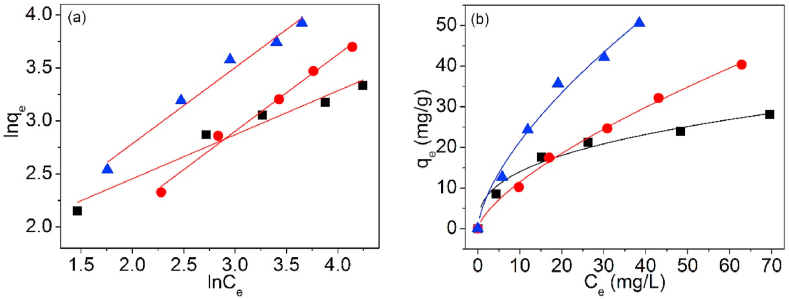
Freundlich isothermal model of UCSBC (■), Zn-modified (●) and Mn-modified (▲) MCSBCs for Pb(II) adsorption, (a) linear fitting, (b) non-linear fitting.

The testing data of Pb(II) adsorption on UCSBC, Zn-modified and Mn-modified MCSBCs were modeled using Langmuir and Freundlich isotherms. The curves are exposed in Fig. 18, Fig. 19, and the model information are tabularized in Table 4.

**Table 4:** Two-parameter isotherm parameters of biochars using linear and non-linear fitting.

Model	Fitting	Parameter	UCSBC	Zn-modified	Mn-modified
Langmuir	Linear	q_cal_ (mg/g)	32.308	84.249	98.792
		K_L_ (L/mg)	0.0760	0.0143	0.0267
		R^2^	0.988	0.965	0.949
	Non-linear	q_cal_ (mg/g)	31.653	86.547	93.666
		K_L_ (L/mg)	0.0808	0.0137	0.0295
		R^2^	0.992	0.997	0.993
Freundlich	Linear	K_fr_ (mg^1-1/n^⋅L^1/n^/g)	5.077	2.076	3.855
		n	2.410	1.381	1.392
		R^2^	0.950	0.988	0.971
	Non-linear	K_fr_ (mg^1-1/n^⋅L^1/n^/g)	6.076	2.343	4.875
		n	2.755	1.449	1.555
		R^2^	0.954	0.993	0.972
Temkin	Linear	b_T_ (J/mol)	367.644	156.497	126.560
		B_T_ (mg/g)	6.742	15.839	19.586
		K_T_ (L/mg)	0.847	0.180	0.313
		R^2^	0.988	0.971	0.985
D–R	Linear	Q_D-R_/(mg/g)	23.306	32.912	42.959
		K_D-R_/(mol^2^/kJ^2^)	3.829	21.346	8.247
		E_m_/(kJ/mol)	0.361	0.153	0.246
		R^2^	0.881	0.848	0.886

In Table 4, for Langmuir and Freundlich isothermals, the R^2^ values of linear fitting were all lower than those of non-linear one, suggesting that non-linear fitting is more proper to designate Pb(II) adsorption on UCSBC, Zn-modified and Mn-modified MCSBCs. Furthermore, the R^2^ values of Langmuir isothermal model via non-linear fitting were all greater than those of Freundlich isothermal model via non-linear fitting, validating that Pb(II) sorption onto UCSBC, Zn-modified and Mn-modified MCSBCs followed the Langmuir isothermal model, i.e., Pb(II) adsorption on UCSBC, Zn-modified and Mn-modified MCSBCs were chiefly administered by monolayer manners, which will be proved by separation factor as discussed below. The cause can be assigned to the effect of ionic groups onto the surface of modified BC.

It should be stressed that for Langmuir isotherm model, its suitability can be additional verified by separation factor (α_L_) [46,47].

(11) $$\alpha_{L}=\frac{1}{\left(1+K_{L}C_{i}\right)}$$

It was reported [46,47] that if the α_L_ values are placed within the region of 0–1, it will enhance the sorption of metal ions onto the surface of an adsorbent. Otherwise, the adsorption on such an adsorbent will be prevented.

Toward examine the suitability of Pb(II) sorption on UCSBC, Zn-modified and Mn-modified MCSBCs, the α_L_ data were weighed on the basis of Langmuir coefficient K_L._ These information are recorded in Table 5.

**Table 5:** The calculated α_L_ data based on Langmuir coefficient K_L_.

C_i_ (mg/L)	Biochar		
	UCSBC	Zn-modified	Mn-modified
20	0.396	0.777	0.651
40	0.247	0.636	0.483
60	0.179	0.538	0.384
80	0.141	0.466	0.318
100	0.116	0.411	0.272

As in Table 5, the α_L_ data were all situated in the scope of 0–1. Based on these information, it is easily concluded that Pb(II) sorption on UCSBC, Zn-modified and Mn-modified MCSBCs can be clarified through Langmuir isotherm model.

##### 3.7.1.3 Temkin isotherm model

Temkin isotherm model [39,40] is usually employed to evaluate the sorption heat, and it can be linearly written as Eq. (12),

(12) $$q_{e}=\frac{RT}{b_{T}}\ln\left(K_{T}C_{e}\right)=B_{T}\;\ln\left(K_{T}C_{e}\right)=B_{T}\;\ln\;K_{T}+B_{T}\;\ln\;C_{e}$$

In Eq. (12), b_T_ (J/mol) is the Temkin coefficient, which is the variant of sorption energy. K_T_ (L/mg) is the balance coefficient and connects with the maximal binding energy, it usually is interrelated to the adsorption heat. B_T_ (mg/g) can be calculated by RT/b_T_.

**Fig. 20:**
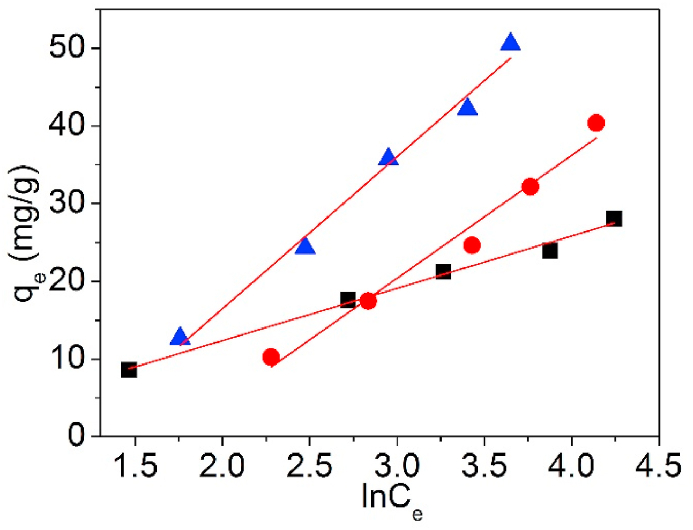
Temkin model of UCSBC (■), Zn-modified (●) and Mn-modified (▲) MCSBCs for Pb(II) adsorption.

Fig. 20 is the Temkin model for Pb(II) adsorption on UCSBC, Zn-modified and Mn-modified MCSBCs. The coefficients are recorded in Table 4.

As manifested in Table 4, the adsorption energy b_T_ of UCSBC, Zn-modified and Mn-modified MCSBCs were all below than 8 kJ/mol. It was stated [47,48] that as the b_T_ value was lower than 8 kJ/mol, the adsorption process is regarded as a physical mode. Consequently, Pb(II) adsorption on UCSBC, Zn-modified and Mn-modified MCSBCs was a physical manner, such as ion exchange or electrostatic attraction.

##### 3.7.1.4 D–R isotherm model

To realize the adsorption free energy and evaluate the adsorption behaviors of Pb(II) adsorption onto UCSBC, Zn-modified and Mn-modified MCSBCs, the experimental data were scrutinized by D–R isotherm model. D–R isotherm model [39,40] can be linearly expressed as Eq. (13a), (13b). While, the adsorption free energy, E_m_ (kJ/mol), can be computed by Eq. (14).

(13a) $$q_{e}=Q_{D-R}\;\exp\left(-K_{D-R}{\left[RT\;\ln\left(1+\frac{1}{C_{e}}\right)\right]}^{2}\right)$$

or,

(13b) $$\ln\;q_{e}=\ln\;Q_{D-R}-K_{D-R}R^{2}T^{2}{\left[\ln\left(1+\frac{1}{C_{e}}\right)\right]}^{2}$$

(14) $$E_{m}=\frac{1}{\sqrt{2K_{D-R}}}$$

In Eqs. (13a), (13b), (14), *Q*_D-R_ (mg/g) is the maximal adsorption amount of metal ions on a sorbent, and K_D-R_ (mol^2^/kJ^2^) is the D–R model parameter.

**Fig. 21:**
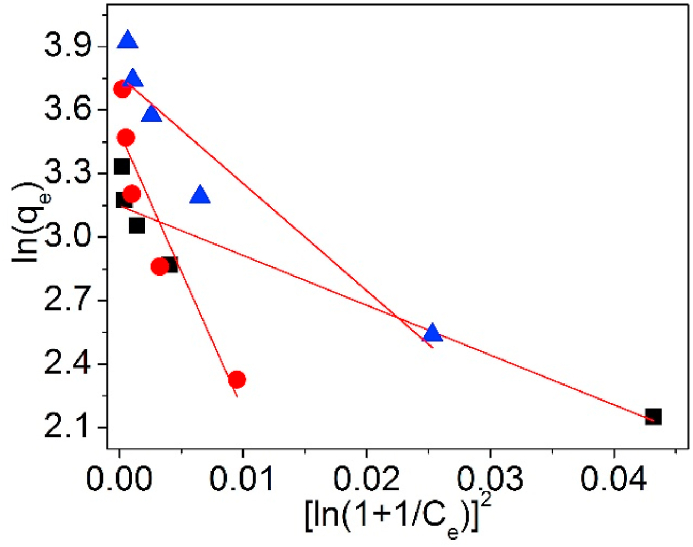
D–R model of UCSBC (■), Zn-modified (●) and Mn-modified (▲) MCSBCs for Pb(II).

Fig. 21 is the D–R model for Pb(II) adsorption on UCSBC, Zn-modified and Mn-modified MCSBCs and the D–R model parameters are programmed in Table 4.

In Table 4, the experimental results fitted poor with D–R isotherm (*R*^2^ values are <0.9). Besides, the *Q*_DR_ data are all lower than those from Langmuir model. Moreover, the E_m_ (kJ/mol) values are sited within the district of 0–0.4 kJ/mol, which is highly lower than 8.0 kJ/mol. It was reported [47,48] that for D-R model, if the adsorption energy calculated is < 8 kJ/mol, such an adsorption process is a physical manner. Based on this result, it can be reasoned out that Pb(II) adsorption on UCSBC, Zn-modified and Mn-modified MCSBCs is a physical style. This outcome is consistent with the conclusion acquired from 1st-O kinetic model.

#### 3.7.2 Three-parameter isotherm model

The theoretical fitting of experimental data through two-parameter isotherm models was a well-accepted method by researchers and readers, such a method will provide an effective way to judge the correctness of experiment result and determine the possible adsorption process. While, the fitting through the two-parameter isotherm models is relatively simple and easier gained. However, the testing results fitted by three-parameter isotherm models maybe more precise than those by two-parameter ones. Hence, for comparison, in our case, the testing data were also fitted with three-parameter isotherms.

##### 3.7.2.1 Sips isotherm model

Sips isotherm model [[43], [44], [45]] has three model parameters and is regarded as the union of Langmuir and Freundlich isotherms (i.e., Langmuir-Freundlich model). It is expressed as Eq. (15).

(15) $$q_{e}=\frac{K_{S}C_{e}^{\beta_{S}}}{1+\alpha_{S}C_{e}^{\beta_{S}}}$$

In Eq. (15), K_S_ (L/mg) and α_S_ are the Sips model constants. β_S_ (dimensionless) is the Sips modeling exponent and is limited in the range of 0–1. It was reported [[43], [44], [45]] that if β_S_ is < 1, Sips isotherm model is closer to Freundlich. In this case, the adsorption process is considered as a heterogeneous style. If not, as β_S_ is closer to 1, Sips isotherm model will be changed as the formation of Langmuir. In this case, the adsorption will be turned into a homogeneous process.

**Fig. 22:**
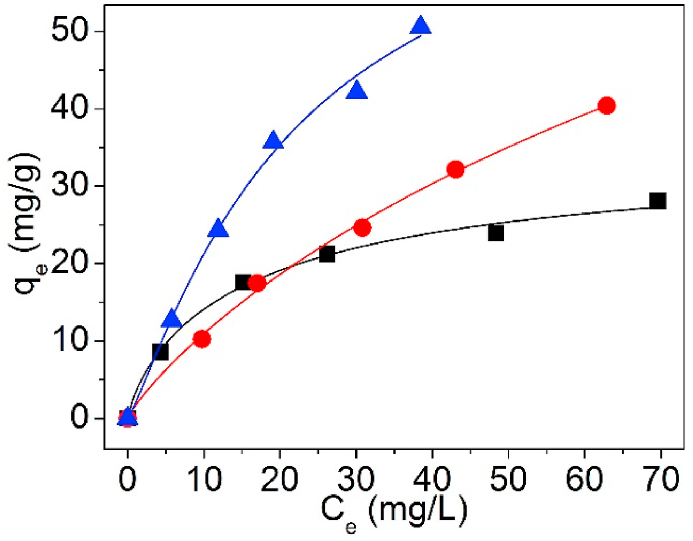
Sips isotherms of UCSBC (■), Zn-modified (●), Mn-modified (▲) MCSBCs for Pb(II).

Fig. 22 is the matching curves of Sips isotherm model of UCSBC, Zn-modified and Mn-modified MCSBCs for Pb(II) by non-linear fitting. The parameter information are presented in Table 6.

**Table 6:** Three-parameter isotherm data of UCSBC, Zn-modified and Mn-modified MCSBCs for Pb(II).

Model	Fitting	Parameter	Biochar		
			UCSBC	Zn-modified	Mn-modified
Sips	Non-linear	Ks	3.560	1.654	1.977
		αs	0.0988	0.0123	0.0262
		βs	0.813	0.857	1.174
		R^2^	0.992	0.997	0.992
R–P	Non-linear	K_RP_	3.477	1.547	2.548
		α_RP_	0.193	0.0899	0.0132
		β_RP_	0.871	0.664	1.178
		R^2^	0.993	0.997	0.992
Toth	Non-linear	K_T_	38.680	245.780	72.108
		α_T_	4.490	13.274	110.753
		n_T_	0.669	0.518	1.391
		R^2^	0.992	0.997	0.992

As showed in Fig. 22 and Table 6, the experimental data matched better with Sips isotherm model (i.e., R^2^ = 0.992, 0.997 and 0.992 for UCSBC, Zn-modified and Mn-modified MCSBCs, respectively). Meanwhile, the β_S_ values of UCSBC, and Zn-modified MCSBC were all lower than 1.0 (i.e., β_S_ = 0.813, and 0.857, respectively). These findings revealed that Pb(II) adsorption on UCSBC, and Zn-modified MCSBC obeyed Sips isotherm model. But, the β_S_ value of Mn-modified MCSBC (i.e., β_S_ = 1.174) was outside the limitation scope of model exponent. Such a trend implies that Sips isotherm model can't be utilized to interpret the sorption activity of Pb(II) on Mn-modified MCSBC. The cause possibly is correlated to the surface modification of KMnO_4_.

##### 3.7.2.2 R–P isotherm model

R–P isotherm model [[43], [44], [45]] is regarded as the extension of Langmuir isotherm model, which is written as Eq. (16).

(16) $$q_{e}=\frac{K_{RP}C_{e}}{1+\alpha_{RP}C_{e}^{\beta_{RP}}}$$

In Eq. (16), K_RP_ and α_RP_ are the R–P model constants. β_RP_ (dimensionless) is as the heterogeneity of the binding surface and was restricted in the scope of 0–1. As stated [49,50], if β_RP_ is approach to 1, R–P isotherm model will be changed as the form of Langmuir model. Contrarily, if β_RP_ approaches to 0, the R–P isotherm model will be renewed as the form of Henry rule.

**Fig. 23:**
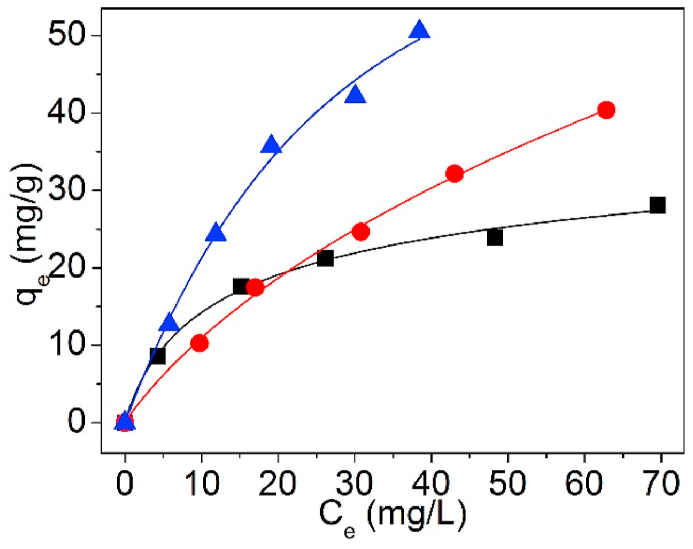
R–P isotherm model of UCSBC (■), Zn-modified (●) and Mn-modified (▲) MCSBCs for Pb(II) adsorption.

The R–P isotherm model of UCSBC, Zn-modified and Mn-modified MCSBCs for Pb(II) was fitted and is offered in Fig. 23. The parameters are listed in Table 6.

As exhibited in Fig. 23 and Table 6, the experimental data fitted well with R–P isotherm model (R^2^ was 0.993, 0.997 and 0.992 for Pb(II) sorption on UCSBC, Zn-modified and Mn-modified MCSBCs, separately). Nevertheless, the β_RP_ figures of UCSBC, and Zn-modified MCSBC were all lower than 1 (i.e., β_RP_ = 0.871, and 0.664, individually). Therefore, the adsorption behaviors of Pb(II) on UCSBC, and Zn-modified MCSBC can be clarified by means of R–P isotherm model. Namely, Pb(II) sorption on UCSBC and Zn-modified MCSBC followed the extension of Langmuir model. Conversely, the β_S_ value of Mn-modified MCSBC (i.e., β_S_ = 1.178) was beyond the scope of model exponent. Thus, it is easy to judge that the Pb(II) adsorption on Mn-modified MCSBC didn't obey R–P isotherm model.

##### 3.7.2.3 Toth isotherm model

Toth isotherm model [[43], [44], [45]] implies the sorption of metal ions arises on the heterogeneous atmosphere. It can be stated as Eq. (17).

(17) $$q_{e}=\frac{K_{T}C_{e}}{{\left(\alpha_{T}+{C_{e}}^{n_{T}}\right)}^{1/n_{T}}}$$

In Eq. (17), K_T_ (L/mg) and α_T_ are the Toth model constants. n_T_ (dimensionless) signifies the heterogeneity of adsorption process. Commonly, n_T_ is restricted the scope of 0–1. It was reported [[43], [44], [45]] that if n_T_ = 1, Toth isotherm model will be transformed as the formation of Langmuir model. In this case, sorption of metal ions will happen onto a homogenous surface. If n_T_ is < 1, the adsorption manner will befall on the heterogeneous system.

**Fig. 24:**
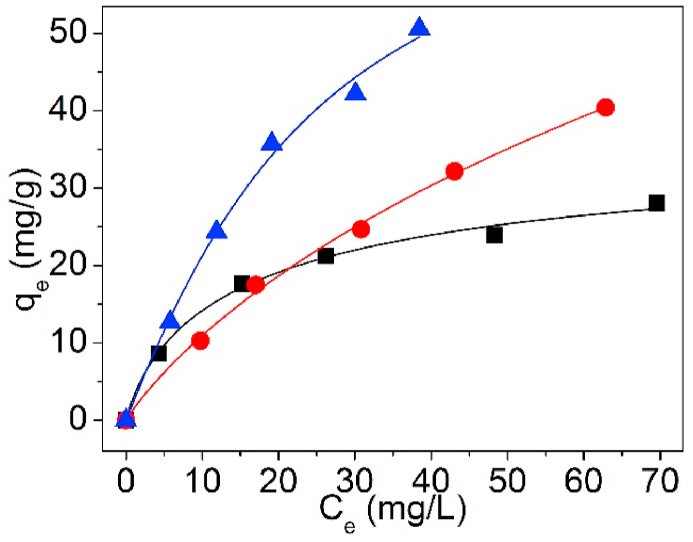
Toth isotherm model of UCSBC (■), Zn-modified (●) and Mn-modified (▲) MCSBCs for Pb(II) adsorption.

Toth isotherm model of UCSBC, Zn-modified and Mn-modified MCSBCs for Pb(II) is presented in Fig. 24. The factors are registered in Table 6.

In Fig. 24 and Table 6, the experimental data matched better with Toth isotherm model (R^2^ = 0.992, 0.997 and 0.992 for Pb(II) sorption on UCSBC, Zn-modified and Mn-modified MCSBCs, separately). Besides, the model factor n_T_ values of UCSBC and Zn-modified MCSBC were situated at 0.669, and 0.518, respectively. These data were placed into the region of 0–1. Consequently, Pb(II) adsorption on UCSBC and Zn-modified MCSBC abided by Toth isotherm model. That is to say, Pb(II) adsorption on UCSBC and Zn-modified MCSBC mainly happened on the heterogeneous surface, rather than a homogenic one. In contrast, the n_T_ value of Mn-modified MCSBC was equal to 1.391 and outside the range of 0–1. Obviously, Pb(II) adsorption on Mn-modified MCSBC didn't occur on the heterogeneous system. Thus, multiple effects will be the leading manner.

### 3.8 Thermodynamic parameters

On the basis of the relationship of adsorption amount against solution temperature, thermodynamic parameters [[46], [47], [48], [49]] can be computed by Eqs. (18), (19), (20).

(18) $$K_{L}=\frac{C_{s}}{C_{e}}$$

(19) $$\ln\left(K_{L}\right)=\frac{\Delta S}{R}-\frac{\Delta H}{RT}$$

(20) $$\Delta G=-RT\;\ln\left(K_{L}\right)$$

In Eqs. (18), (19), (20), K_L_ is the equilibrium partition coefficient. ΔG (J/mol) is the change in free energy, ΔH (J/mol) is the enthalpy change, and ΔS (J/mol·K) is the entropy change. While, R is the molar gas constant (R = 8.314 J/mol·K), T (K) means the absolute temperature, and K_L_ is the partition coefficient at balance state. In addition, C_e_ (mg/L) is the sorption amount of metal ions at stability state after sorption, C_s_ (mg/L) denotes the residual amount of metal ions in solution after sorption.

**Fig. 25:**
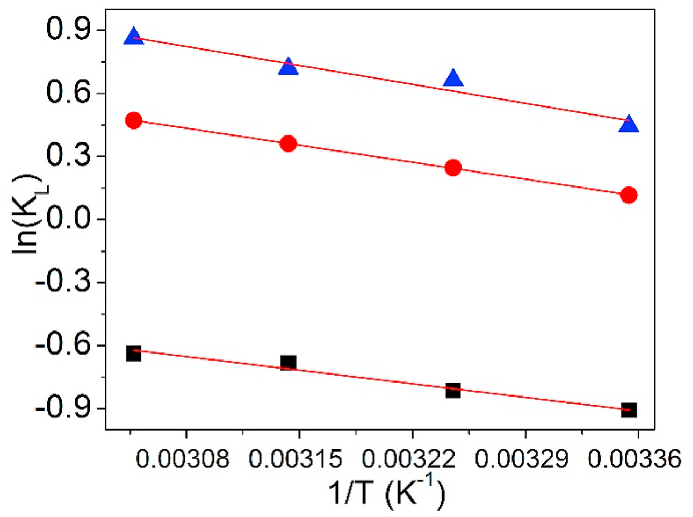
Thermodynamic model of Pb(II) adsorption on UCSBC (■), Zn-modified (●) and Mn-modified (▲) MCSBCs.

To decide the thermodynamic information of UCSBC, Zn-modified and Mn-modified MCSBCs for Pb(II), thermodynamic model was established and is displayed in Fig. 25 and the thermodynamic parameter information are tabularized in Table 7.

**Table 7:** Relevant parameters of the thermodynamic model.

Biochar	T (K)	ΔG (KJ/mol)	ΔS (J·K/mol)	ΔH (KJ/mol)	R^2^
UCSBC	298.15	2.277	18.236	7.681	0.962
	308.15	2.110			
	318.15	1.826			
	328.15	1.761			
Zn-modified MCSBC	298.15	−0.291	33.178	9.600	0.999
	308.15	−0.638			
	318.15	−0.965			
	328.15	−1.300			
Mn-modified MCSBC	298.15	−1.113	39.785	10.695	0.933
	308.15	−1.719			
	318.15	−1.923			
	328.15	−2.380			

As in Table 7, for Pb(II) adsorption on UCSBC, Zn-modified and Mn-modified MCSBCs, the values of ΔS were positive, i.e., ΔS >0, indicating that Pb(II) sorption onto UCSBC, Zn-modified and Mn-modified MCSBCs met entropy increase principle and the sorption process was irreversible. Moreover, the data of ΔH were all positive, demonstrating that Pb(II) adsorption on UCSBC, Zn-modified and Mn-modified MCSBCs was an endodermal process. Elevating the temperature of solution will be favorable to Pb(II) adsorption, which is agreement with the observation of adsorption amount versus solution temperature. Furthermore, the negative ΔG values of Zn-modified to Mn-modified MCSBCs evidenced that Pb(II) adsorption on them was a spontaneous process. Note that, ΔG values of UCSBC were all positive in the temperature region of 25–55 °C, suggesting that Pb(II) adsorption on UCSBC wasn't a spontaneous process and such a process will be a spontaneous process as the temperature was elevated. In contrast, the ΔG values of both Zn-modified and Mn-modified MCSBCs for Pb(II) were all negative. These results implies that the modification of UCSBC through KMnO_4_ and ZnCl_2_ can increase its adsorption for Pb(II), designating excellent modification effects of UCSBC by inorganic modifiers. This finding provides an effective route for the modification of waste coconut shell and exhibits promising application in the handling of metal-containing wastewater.

**Fig. 26:**
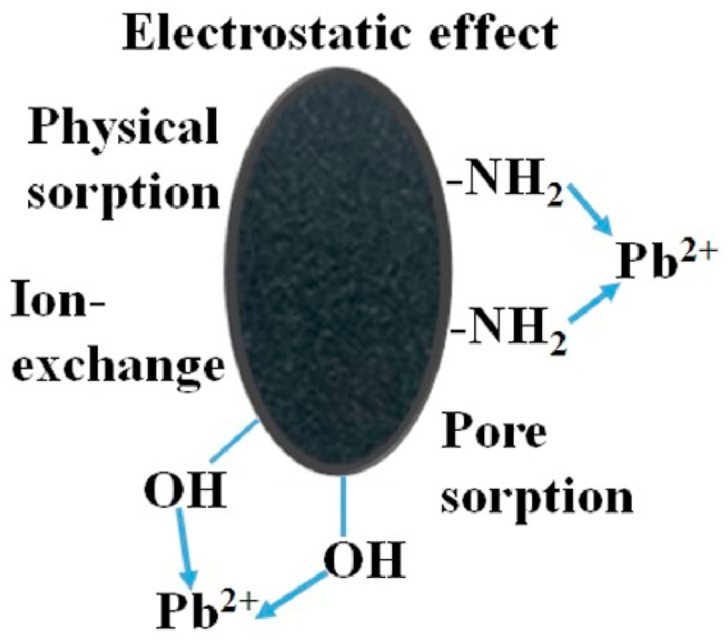
The proposed adsorption mechanism.

Based the fore-mentioned results, the adsorption mechanism can be schematically illustrated as Fig. 26.

### 3.9 A comparison of various sorbents for Pb(II)

To evaluate the adsorption of Zn- and Mn-modified MCSBCs for Pb(II), a comparison between them with various adsorbents is tabulated in Table 8.

**Table 8:** A comparison of Langmuir q_m_ of various sorbents for Pb(II) in references.

Adsorbent	q (mg/g)	pH	Temperature (^o^C)	References
Cellulose hydrogel	916.26	–	30	[18]
Silica Aerogel/Quince seed mucilage	37	4	rt	[25]
Avocado leaf adsorbent	60.46	3	25	[26]
Natural kaolin	4.24	6.87	25	[30]
EDTA-modified chitosan/magnetic biochar	163.19	3	45	[34]
Co-pyrolysis biochar from fresh food waste and rice husk	245.52	5.5	25	[39]
MnO_2_-modified magnetic biochar	49.64	7.93	30	[45]
cellulose-based absorbent	164	2.6	30	[50]
UCSBC	31.653	5	25	This study
Zn-modified MCSBC	86.547		25	
Mn-modified MCSBC	93.666		25	

Noticeably, Zn- and Mn-modified MCSBCs have higher q values than some other adsorbents, suggesting that MCSBCs can be potentially used to remove Pb(II) from water.

## 4 Conclusion

The modified coconut shell biochars (MCSBCs) by ZnCl_2_ and KMnO_4_ were developed and their adsorptions for Pb(II) were explored. Based on linear and non-linear fitting methods, kinetic and two-parameter and three-parameter isotherms were used to evaluate the experiment data. The following results can be achieved.

(1) The Langmuir maximal adsorption amounts of the UCSBC, Zn-modified and Mn-modified MCSBCs could be arrived at 31.653, 86.547 and 93.666 mg/g, respectively.
(2) The non-linear fitting of kinetic models revealed that Pb(II) adsorption on the UCSBC, Zn-modified and Mn-modified MCSBCs followed Lagergren first-order and Avrami fractional- order kinetic models.
(3) Two-parameter and three-parameter isotherm models evidenced that Pb(II) adsorption on UCSBC, Zn-modified and Mn-modified MCSBCs obeyed the Langmuir and Sips isotherm models.
(4) Thermodynamic parameters proved that the modification of UCSBC via KMnO_4_ and ZnCl_2_ can efficiently increase its adsorption amount for Pb(II). While, it was ascertained that Pb(II) adsorption on the modified Zn-modified and Mn-modified MCSBCs was a spontaneous process. Conversely, Pb(II) sorption on the un-modified CSBC wasn't a spontaneous process and elevating temperature will change such a sorption manner.

## Data availability statement

The authors declare that the data supporting the findings of this study are available within the paper, for more detailed data should be sent to the corresponding authors on reasonable request.

## CRediT authorship contribution statement

**Jingyi Chen:** Formal analysis, Data curation, Conceptualization. **Qianqian Duan:** Validation, Investigation, Data curation. **Chunyu Ji:** Investigation, Formal analysis, Data curation. **Junsheng Liu:** Writing – review & editing, Supervision, Conceptualization. **Ziyao Wang:** Validation, Investigation, Formal analysis. **Jiahui Song:** Methodology, Formal analysis. **Wei Li:** Formal analysis, Data curation. **Chaojian Zhang:** Writing – original draft, Investigation, Formal analysis.

## Declaration of competing interest

The authors declare that they have no known competing financial interests or personal relationships that could have appeared to influence the work reported in this paper.
